# Growth rate trades off with enzymatic investment in soil filamentous fungi

**DOI:** 10.1038/s41598-020-68099-8

**Published:** 2020-07-03

**Authors:** Weishuang Zheng, Anika Lehmann, Masahiro Ryo, Kriszta Kezia Vályi, Matthias C. Rillig

**Affiliations:** 1 PKU-HKUST ShenZhen-Hong Kong Institution, Shenzhen, 518057 China; 2 Plant Ecology, Institut für Biologie, Freie Universität Berlin, Altensteinstr. 6, 14195 Berlin, Germany; 3 Berlin-Brandenburg Institute of Advanced Biodiversity Research (BBIB), 14195 Berlin, Germany

**Keywords:** Microbial ecology, Fungal ecology

## Abstract

Saprobic soil fungi drive many important ecosystem processes, including decomposition, and many of their effects are related to growth rate and enzymatic ability. In mycology, there has long been the implicit assumption of a trade-off between growth and enzymatic investment, which we test here using a set of filamentous fungi from the same soil. For these fungi we measured growth rate (as colony radial extension) and enzymatic repertoire (activities of four enzymes: laccase, cellobiohydrolase, leucine aminopeptidase and acid phosphatase), and explored the interaction between the traits based on phylogenetically corrected methods. Our results support the existence of a trade-off, however only for the enzymes presumably representing a larger metabolic cost (laccase and cellobiohydrolase). Our study offers new insights into potential functional complementarity within the soil fungal community in ecosystem processes, and experimentally supports an enzymatic investment/growth rate trade-off underpinning phenomena including substrate succession.

## Introduction

Soil saprobic fungi drive essential ecosystem processes including decomposition and soil aggregation^1,2^. In order to understand contributions of fungi to such processes it is helpful to adopt a functional traits approach^3^. Two traits are expected to be of great importance for describing essential functions of fungi in soil: growth rate, because it denotes how fast and to what extent fungi encounter their substrate^4^; and enzymatic repertoire, since it describes the ability to break down and utilize various substrates^5,6^.

Traditional concepts in mycology have long assumed a fundamental trade-off between growth rate and enzymatic abilities^7^. For example, substrate successions have been described as a shift in fungal strategies, where the ability to grow fast and the ability to use more available carbon compounds change from early to later stages of succession^8,9^. For ectomycorrhizal fungi, initial experimental evidence ^10^ and field observations^11^ have suggested a trade-off between investment in enzymes and competitiveness or exploration distance. Moeller and Peay showed an inverse relationship between competitiveness and enzymatic activity using pine seedlings associated with fungi from three genera^10^. Agerer suggested that ectomycorrhizal fungi that cannot produce phenoloxidases to decompose lignin had to use more long-distance exploration for easily decomposed carbon resources, and vice versa^11^. Nevertheless, to our knowledge, the existence of such a trade-off has never been directly experimentally addressed for any group of soil fungi. Soil fungi, either free-living or associated in symbiosis with host plants, contribute to the terrestrial carbon cycle by organic matter decomposition and mycelium biomass^12,13^. The fungal enzyme repertoire and growth rate, and hence the ability to utilize readily available carbon sources, or substrates that are more difficult to degrade, influences the turnover of soil carbon, and thus the release of carbon from soil to the atmosphere. Hence, it is highly relevant to build on the pioneering work on trade-offs in fungal enzyme activity and mycelium growth rate by providing a specific experimental test.

For this, we asked in experiments if and how growth rate and enzymatic repertoire are linked. We compiled a set of 31 fungal strains, isolated from the same soil and covering three fungal phyla (Ascomycota, Basidiomycota and Mucoromycota). This suite comprises culturable strains which are abundant in their ecosystem and exhibit a broad versatility in their trait expressions^14,15^. For these 31 strains, we investigated enzymatic activity of four enzymes. These enzymes are commonly used in studies investigating fungal enzyme repertoires because they represent fungal capability to acquire carbon, nitrogen and phosphorus from organic matter decomposition^5,6,16^. The enzymes were laccase (Lac; EC 1.10.3.2) and cellobiohydrolase (Cel; EC 3.2.1.91), enzymes involved in the degradation of two major and abundant recalcitrant carbon forms, lignin and cellulose, respectively; leucine aminopeptidase (Leu; EC 3.4.11.1) that hydrolyzes leucine and other nonpolar amino acids, and therefore is commonly measured as an indication of nitrogen-related enzyme activity; and acid phosphatase (Pho; EC 3.1.3.2) that releases free attached phosphoryl groups, a key step in fungal P uptake.

## Materials and methods

### Fungal materials

The filamentous saprobic fungi used in our experiments were isolated (at room temperature, 22 °C, i.e. at the temperature we also use in the experiments here) from soil samples collected in one ecosystem, a grassland at Oderhänge Mallnow (Germany, 52° 27.778′ N, 14° 29.349′ E). The detailed information about isolation method, strain deposit and strain identification has been published^17^. Briefly, the isolation procedure focused on spores and mycelium from soil and hyphae. Soil samples were washed and diluted to reduce the abundance of spores and with it the likelihood of heavy sporulators among the isolates. Prepared soil suspensions were added to a variety of growth media supplemented with antibiotics favoring the growth of Ascomycota, Basidiomycota and Mucoromycota but suppressing bacterial colonization. The final fungal set was recruited from isolates establishing viable colonies on potato dextrose agar (PDA; Carl Roth GmbH) at room temperature (22 °C). The 31 fungal strains comprise 20 Ascomycota, 4 Basidiomycota and 7 Mucoromycota (Fig. S1, Table S1, Table S2).

### Experimental setting

Fungi were grown in Petri dishes with potato dextrose agar (PDA): this is a rich medium containing 20 g dextrose and 4 g potato extract per liter. In PDA, all 31 fungi can grow without overt nutrient limitation, and fungal strains can also invest in enzyme production^18^. Furthermore, we did not use lignin or other substances to trigger the activity of specific enzymes. We placed sterilized cellophane membranes (#165-0963, Bio-RAD, USA) on the agar surface to facilitate extraction of media-free mycelia, since cellophane membranes prevent mycelia from penetrating into agar, and are easily separable from the mycelia^16^. We grew fungi from mycelium plugs at room temperature (22 ˚C) until the cultivation periods were past half of their linear growth phase. The cultivation periods (Supplementary Table S2) were determined by the growth curves measured in a preliminary experiment under similar conditions (data not shown). Six replicates were prepared for each strain resulting in 186 experimental units.

### Colony radial growth rate (K_r_)

All colonies were imaged (Epson Perfection V700) at two time points. In each picture, the radius was measured in triplicate with Image-J (1.49 v23; 18), and subsequently averaged to calculate the growth rate (μm·h^−1^), following the equation: *K*_*r*_ = (*radius*_*t2*_* − radius*_*t1*_)/(*t*_*2*_* − t*_*1*_).

### Enzymes

We profiled fungal enzymatic activities of laccase (Lac; lignin degradation), cellobiohydrolase (Cel; cellulose degradation), leucine aminopeptidase (Leu; catalyzes the hydrolysis of peptides) and acid phosphatase (Pho; releases free attached phosphoryl groups). Laccase and cellobiohydrolase target less available nutrients and hence may have a higher metabolic cost, while leucine aminopeptidase and acid leucine aminopeptidase target readily available nutrients may have a lower metabolic cost. The profiling was done by a microplate photometric method following a modified protocol by Courty et al.^20^. From each petri dish, we cut 8 small pieces of mycelium (3–5 mm^2^; 2 subsample × 4 enzymes) from the colony’s peripheral zone, and weighed this immediately to get fresh weight (FW). Each piece was stored in an Eppendorf tube at 4 °C and processed within 24 h. The details of the measuring conditions are in Supplementary Table S3. One unit (U) of each enzyme activity was defined as the amount of the enzyme releasing 1 µmol of the corresponding substrate per min. Results of enzymatic tests were standardized by the dry weight (DW) of the mycelial sample. We obtained the DW of the small pieces by converting the fresh weight (FW) following the equation: *DW* = *FW* × *ratio*_*d/f*_, where *ratio*_*d/f*_ is the biomass dry/fresh ratio. This ratio was calculated based on a quarter of the colony which was removed at the same time as the small pieces for the enzyme measurements. First, we measured this quarter’s fresh weight, then dried it at 45 °C over night, weighed again, and calculated the *ratio*_*d/f*_. The standardized enzyme activity unit is expressed as: U·mg_(dw)_^−1^. To obtain more accurate enzymatic data, one can add catalase to remove the potential contribution of peroxidase. However, our goal was to compare patterns among the different species, so we see our interpretation unaffected.

### Statistics

The relationship between *K*_*r*_ and enzyme activities was tested (i) considering single enzymes and (ii) as a joint effect of all the enzymes. Data were transformed as needed to meet parametric statistical assumptions. First, before exploring the relationship between growth rate and enzymatic repertories, we tested for phylogenetic signals in the trait data, since one concern is that the relatedness found between traits could arise from phylogenetic dependency^21^. This means that closely related species may display similar trait values due to their common ancestry, which violates the statistical assumption on independent sampling. Therefore, we tested whether the traits had phylogenetic signals based on Blomberg’ K statistics^21^ using the R package “picante”^22^ and if trait data had to be phylogenetically corrected. The different sample size within the three phyla does affect any analysis with phylum as explanatory variable; but this can be corrected by application of type III sums of squares. However, uneven sampling will not change the phylogenetic signal of traits, because the calculation is based on the distance between phylogenetic tree tips and the difference of corresponding trait data. Second, we applied linear regression on *K*_*r*_ and log transformed enzyme activities and further validated our findings by quantile regression with the R package “quantreg” (https://github.com/cran/quantreg). Due to unmeasured factors having the potential to limit fungal growth besides the targeted enzyme activities (leading to a ‘wedge’-shaped data distribution), it is advisable to investigate the relationship estimates at the maxima rather than at the mean of the response distribution^23,24^. Third, we used the first principal component (PC1), extracted from PCA applied on 4 enzyme activities, to represent the joint effects of enzymes, and regressed it with *K*_*r*_. The sample size (n) is 31, representing the tested fungal species means. All analyses were conducted in R 3.6.2^25^. Raw data and R code are archived in figshare (https://figshare.com/articles/Zheng_et_al_biomass_enzyme_tradeoff/11842767).

## Results and discussion

**Figure 1:**
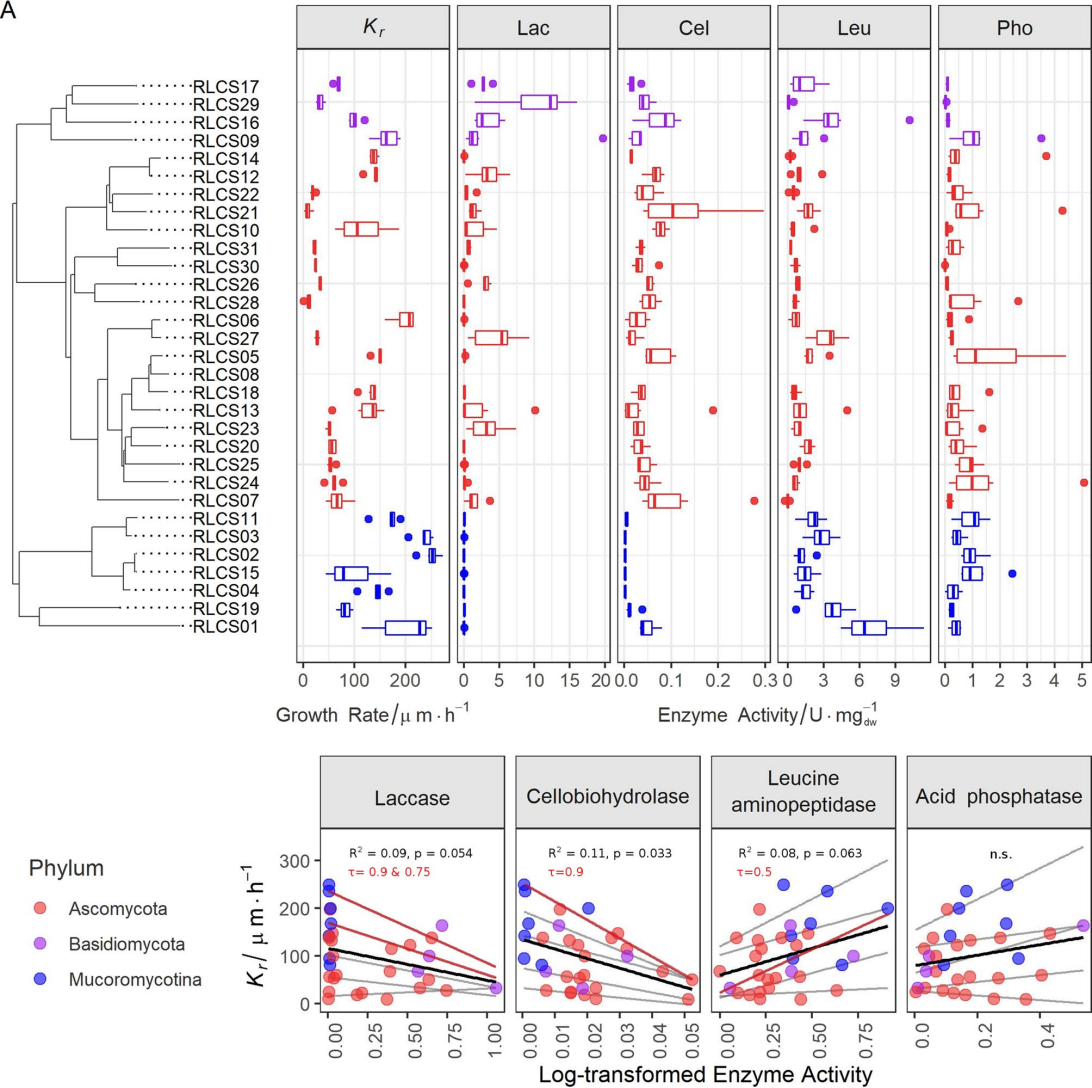
Traits of 31 saprobic fungi and the interaction between colony radial growth rate (*K*_*r*_ in µm·h^−1^) and activities of laccase (Lac in unit·mg^−1^ dry weight), cellobiohydrolase (Cel in unit·mg^−1^ dry weight), leucine aminopeptidase (Leu in unit·mg^−1^ dry weight) and acid phosphatase (Pho in unit·mg^−1^ dry weight). **(A)** Tukey boxplot of traits (n = 6) with whiskers indicating 25th and 75th percentiles; dots beyond the reach of whiskers are potential outliers. **(B)** Regression between *K*_*r*_ and log-transformed enzyme activity data (n = 31). Grey lines are quantile regression lines at the quantiles τ = 0.1, 0.25, 0.5, 0.75 and 0.9, with the significant ones highlighted and annotated in red. The black line represents the linear regression line and its R^2^ and p-value are shown in the plot. The details of the regression statistics can be found in Table S6.

We found high variability in traits across the tested 31 fungi covering 11 orders from three different phyla (Fig. 1A, Fig. S2–S6, Table S1 and S2). The colony radial growth rate (*K*_*r*_) varied from 9.2 to 250.2 µm·h^−1^ with the fastest growing strains found in phylum Mucoromycota (Fig. S2). The enzyme profiles showed that Lac activity was much higher in Basidiomycota than in Ascomycota, while it was absent in Mucoromycota (Fig. 1A; Fig. S3). Mucoromycota had higher Leu compared to the Ascomycota strains (Fig. S5). Within Mucoromycota, Mortierellales strains (RLCS02-04, 11 and 15) did not produce Cel (0.00091 ± 0.00059 U·mg_(dw)_^−1^, n = 5). All strains were positive for Pho activity and had no difference at the phylum level (Fig. S6). These results are broadly consistent with the known enzymatic features of fungal groups^5,6,20^. As a caveat, we note that the use of ABTS (2,2′-azino-bis(3-ethylbenzothiazoline-6-sulfonic acid), a nonspecific substrate for enzyme tests, can lead to overestimation of Lac activity, since it reacts with other peroxidases^26^. At the same time, dextrose from PDA could give rise to catabolite repression that likely affected Cel/Lac enzymatic data^26^.

Before exploring the relationship between growth rate and enzymatic repertoires, we tested the phylogenetic signals of the traits, since one concern is that the relatedness found between traits could arise from phylogenetic constraints^21^. This means, closely related species may display similar trait values due to their common ancestry. Based on the K statistics, we confirmed that in the current setting growth rate *K*_*r*_ did not show phylogenetic signal (Table S4). Therefore, here, phylogeny was not the main driver for explaining the patterns we found.

We found evidence of a trade-off between enzyme activity and growth rate; however, not for all enzymes (Fig. 1B and Table S5). Linear regression analyses revealed that *K*_*r*_ was negatively correlated to Lac (*P* = 0.054, R^2^ = 0.09) and Cel (*P* = 0.033, R^2^ = 0.11), suggesting a trade-off between these variables. To obtain further insight in the nature of the relationship of *K*_*r*_ and Lac and Cel, respectively, we conducted quantile regression analyses. These revealed that the trade-off between growth and enzymes (here, Lac and Cel) was significant at higher quantiles. These quantile regressions are likely more robust than standard linear regression, given the clearly wedge-shaped distribution of data (indicating that other variables also exert an influence on the dependent variable). This can be interpreted to mean that growth rate limits the expression of the maximum enzyme activities that are achievable. On the other hand, Leu positively affected *K*_*r*_, while Pho did not show a clear pattern in either linear or quantile regression (Fig. 1B and Table S6).

This link between growth rate and enzyme activities is an aspect of intrinsic strategy of the fungi, rather than a reaction to their cultivation conditions. In our experiments, the traits were measured during the linear phase when PDA provides sufficient nutrients without enzyme-specific stimuli, since its carbon sources are starch and dextrose (more available C), but not cellulose and lignin (less available C), and the major organic nitrogen is asparagine but not leucine^27^. This means that enzyme expression under this condition is at a basal level rather than highly expressed. Hence the relationship we found likely describes fungal behavior in the beginning of substrate exploration rather than a reaction to a specific substrate.

The trade-off we found suggests that Lac and Cel enzymes come at a higher metabolic cost to the mycelium, leaving less for allocation to growth. This is congruent with the implications of the theoretical model concerning exoenzyme activity and microbial carbon investments and returns^28^. Fungi can be cost inefficient when producing extracellular enzymes, i.e. the return of exoenzymes regarding C and hence metabolic energy is less than the investment for producing them. Additionally, circumstantial evidence suggests that fungi in the functional groups of polymer decomposers and degraders of resistant compounds during later stages of substrate successions are typically associated with strong territoriality and antibiotics production^29^. This may mean that such fungi, in order to ‘protect’ their enzymatic investment, will produce defensive compounds, which likely represent an additional metabolic cost. In our study, *Fusarium* spp. (RLCS05 and RLCS13) and *Chaetomium* sp. (RLCS06) from Ascomycota were typical polymer decomposers, and the Basidiomycete fungi were degraders of resistant compounds. All the genera of polymer decomposers and degraders of resistant compounds used in the study have been reported as antibiotic producers^30–35^, however we did not measure antibiotic resistance in this experiment.

**Figure 2:**
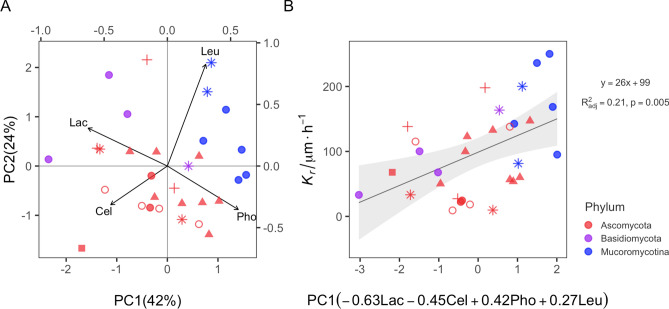
Linear regression between colony radial growth rate (*K*_*r*_) and the joint pattern of enzymes represented by principle component 1 (PC1). **(A)** PCA biplot. **(B)** Linear regression PC1 vs. *K*_*r*_. Color coding represents phyla and different symbols represent different orders.

Under natural conditions, fungi produce a cocktail of enzymes to acquire resources, hence we also explored the impact of the joint effect of the four enzymes on *K*_*r*_. First, we revealed that compared to single enzymes, the overall enzyme activity (reflected in PC1) has a stronger effect on *K*_*r*_ (Fig. 2A). This is especially true for Basidiomycota strains (purple symbols in Fig. 2A; R^2^ = 0.77, F_1,2_ = 11.12; p = 0.08) since a larger PC1 value indicates a lower value of Lac and Cel and a higher value of Pho and Leu. This further supports the hypothesis of a trade-off between growth and enzymatic repertoire differentiated to specific enzymes. To have high *K*_*r*_, a fungus has to invest more into ‘cheap’ enzymes (here: Pho and Leu) for nutrient supply and less in ‘costly’ enzymes (here: Lac and Cel) for energy supply; and vice versa for low growth rates. Even though we measured only a limited number of enzymes, our study presents the first empirical evidence showing effects of enzymes on *K*_*r*_.

In conclusion, we showed that, for saprobic fungi across several phyla, growth rate is related to multiple enzyme activities, and depending on ‘cost efficiency’ of exploiting certain substrates, enzymes are linked positively or negatively with fungal growth rate. Our analysis also highlights the joint effect of the entire enzymatic repertoire, to the extent measured, rather than a key enzyme as responsible for this pattern. Substrate successions are of basic ecological interest since they represent a microbial equivalent of the more well-studied plant succession. The driving force underpinning successions are trade-offs in the life histories of the organisms involved. Our results offer experimental evidence for the existence of a tradeoff in enzymatic investment vs. growth rate, a trade-off that has traditionally been invoked to explain the phenomenon of fungal substrate succession. Our results also indirectly offer new insights into the potential functioning of soil fungal communities: the fungi we examined here came from one community and differed noticeably in the traits we measured. Such difference in traits at the community level could give rise to functional complementarity effects, for example with consequences for ecosystem processes that involve carbon processing, such as decomposition and soil aggregation^36^.

## Supplementary information

Supplementary file1 (PDF 452 kb)

## Data Availability

All data and code have been submitted to figshare, where they are publicly available: https://figshare.com/articles/Zheng_et_al_biomass_enzyme_tradeoff/11842767.
