# Supplementary material for: Growth rate trades off with enzymatic investment in soil filamentous fungi
Source: Sci Rep. 2020 Jul 3;10:11013. doi: 10.1038/s41598-020-68099-8 (PMC7335036; doi:10.1038/s41598-020-68099-8)
Supplement: Supplementary file 1 — Supplementary file1 (PDF 452 kb) [file 41598_2020_68099_MOESM1_ESM.pdf]

Supplementary Information

Growth rate trades off with enzymatic investment in soil filamentous fungi

Weishuang Zheng[1] Anika Lehmann[2,3] Masahiro Ryo[2,3] Kriszta Kezia Vályi[2,3] Matthias C. Rillig[2,3,*]

[1] PKU-HKUST ShenZhen-Hong Kong Institution, Shenzhen 518057, China;

[2] Freie Universität Berlin, Institut für Biologie, Plant Ecology, Altensteinstr. 6, D-14195 Berlin, Germany;

[3] Berlin-Brandenburg Institute of Advanced Biodiversity Research (BBIB), D-14195 Berlin, Germany

* Correspondence to Matthias C. Rillig, matthias.rillig@fu-berlin.de; +49 (0)30 838-53165

1. Fungal material

The filamentous saprobic fungi used in our experiments were isolated from soil samples collected in Oderhänge Mallnow (Germany, 52°27.778'N, 14°29.349'E) – a 304 ha nature conservation area (grassland). The set comprised 31 fungal isolates from the phyla Ascomycota, Basidiomycota and Mucoromycota, which are all saprobic (Fig. S1). The technical details of isolation, identification and phylogenetic tree building have been published (Lehmann et al. (*in press*)). In general, the phylogenetic tree was reconstructed based on the complete intergenic transcribed spacer (ITS) and a part of the large rRNA subunit (LSU). Thereafter, the taxon identifications of strains were determined (Table S1). Fungal strains have been deposited at the German Collection of Microorganisms and Cell Cultures (DSMZ; Table S2).

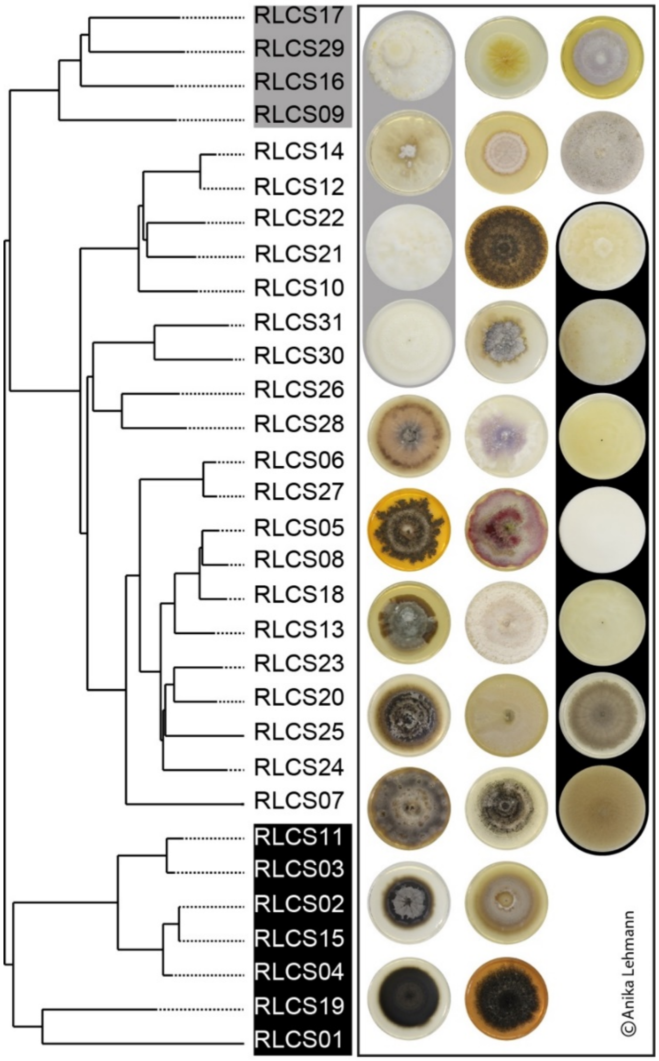

Fig. S1. Phylogenetic (neighbor-joining) tree of the 31 fungal strains belonging to the phyla Ascomycota (white), Basidiomycota (grey) and Mucoromycota (black). Colony pictures are from four-week old cultures grown on potato dextrose agar, and their order follows the order of the phylogenetic tree.

Table S1: Taxonomy of the 31 strains.

Phylum	Class	Order	Family	Taxon identification *	Strain ID
**Basidiomycota**	Agaricomycetes				
		Agaricales			
			Entolomataceae	*Clitopilus* sp.	RLCS17
			Agaricaceae	*Macrolepiota excoriata*	RLCS29
			Pleurotaceae	*Pleurotus pulmonarius*	RLCS16
		Polyporales			
			Coriolaceae	*Trametes versicolor*	RLCS09
**Ascomycota**	Dothideomycetes				
		Pleosporales			
			Didymellaceae	Didymellaceae strain 2	RLCS14
				Didymellaceae strain 1	RLCS12
			Phaeosphaeriaceae	*Paraphoma chrysanthemicola*	RLCS22
			Cucurbitariaceae	*Pyrenochaetopsis leptospora*	RLCS21
			Pleosporaceae	*Alternaria alternata*	RLCS10
	Eurotiomycetes				
		Chaetothyriales			
			Herpotrichiellaceae	*Cyphellophora* sp.	RLCS31
				Exophiala equina	RLCS30
	Leotiomycetes				
		Helotiales			
			Helotiaceae	*Tetracladium marchalianum*	RLCS26
				Tricladium sp.	RLCS28
	Sordariomycetes				
		Sordariales			
			Chaetomiaceae	*Chaetomium angustispirale*	RLCS06
				Thielavia inaequalis	RLCS27
		Hypocreales			
			Nectriaceae	*Fusarium* sp.	RLCS05
				Gibberella tricincta	RLCS08
				Gibberella sp.	RLCS18
				Fusarium solani	RLCS13
			Stachybotryaceae	Stachybotryaceae strain 1	RLCS23
			Ophiocordycipitaceae	*Purpureocillium lilacinum*	RLCS20
			Bionectriaceae	*Hydropisphaera* sp.	RLCS25
			Clavicipitaceae	*Metarhizium marquandii*	RLCS24
		Xylariales			
			Amphisphaeriaceae	Amphisphaeriaceae strain 1	RLCS07
**Mucoromycota**	Mortierellomycetes				
		Mortierellales			
			Mortierellaceae	*Mortierella alpina* strain 2	RLCS11
				Mortierella alpina strain 1	RLCS03
				Mortierella elongata strain 2	RLCS02
				Mortierella elongata strain 1	RLCS15
				Mortierella exigua	RLCS04
	Umbelopsidomycetes				
		Umbelopsidales			
			Umbelopsidaceae	*Umbelopsis isabellina*	RLCS19
	Mucoromycetes				
		Mucorales			
			Mucoraceae	*Mucor fragilis*	RLCS01

*: The best resolved tree annotation passing 80% threshold of bootstrap approach

2. Cultivation periods

Hyphae can grow as fast as few micrometers to millimeters per hour and develop different growth strategies in different phases. In general, we harvest fungal tissue when the individual colony in the late linear growth phase (Table S2). Therefore, for each strain, the duration and onset of the linear growth phase were measured in a preliminary experiment, and the related data is given in Table S2.

Table S2: Growth period in this experiment and preliminary data (columns with asterisks) measured under the same strains growing in petri dishes containing PDA at room temperature. Maximal diameters were recorded when colonies stopped growing or reached the edge of the Petri dish. All the strains are deposited in German Collection of Microorganisms and Cell Cultures GmbH. Ascomycota in white, Basidiomycota in grey and Mucoromycota in black.

Strain ID	Growth period/ day	K_r/ µm h^{-1} *	Time reaching maximal diameter/ day *	Final diameter/ mm *	DSMZ accession number
RLCS17	14	79.5	16	74	DSM100324
RLCS29	33	23.2	35	70	DSM100288
RLCS16	13	84.6	35	85	DSM100408
RLCS09	11	178.6	7	85	DSM100406
RLCS14	26	44.7	35	76	DSM100404
RLCS12	12	129.7	11	85	DSM100405
RLCS22	36	44.6	35	75	DSM100401
RLCS21	12	117.9	9	85	DSM100327
RLCS10	17	152.4	16	85	DSM100286
RLCS31	13	70.5	35	40	DSM100328
RLCS31	36	20.2	35	36	DSM100328
RLCS30	33	20.7	35	44	DSM100291
RLCS26	26	30	35	42	DSM100330
RLCS28	36	25.7	35	51	DSM100323
RLCS06	9	198.5	NA	NA	DSM100400
RLCS27	26	26.8	35	29	DSM100326
RLCS05	11	215.2	16	82	DSM100403
RLCS08	14	183	16	62	DSM100325
RLCS18	13	78.1	35	78	DSM100287
RLCS13	14	125.5	15	80	DSM100290
RLCS23	26	42.6	35	55	DSM101519
RLCS20	13	55.2	35	42	DSM100329
RLCS25	22	31.1	35	78	DSM100292
RLCS24	20	37.6	9	85	DSM100410
RLCS07	13	196.8	35	80	DSM100284
RLCS11	11	148.1	18	85	DSM100289
RLCS03	6	220	11	83	DSM100285
RLCS02	6	247.4	11	85	DSM100407
RLCS15	10	148.5	16	77	DSM100402
RLCS04	10	215.2	16	62	DSM100322
RLCS01	6	372.8	7	85	DSM100293

3. Enzyme activity measurements

In order to profile fungal enzyme activities in the linear growth phase, we collected younger mycelia from the outer zone of the colony. For each enzyme, two small pieces of mycelium (3-5 mm^2) was cut, weighed freshly and stored at 4°C in a 1 ml Eppendorf tube. For each plate, eight pieces of mycelium were prepared for the enzyme measurements, which were conducted within 24 hours. The data present for each unit were the average of two subsamples.

The activities of enzymes were tested by a microplate photometric method.

The acid phosphatase activity was tested based on the hydrolysis of pNPP(para-nitrophenylphosphate, a synthetic substrate) into pNP(para-nitrophenol) + P. The reaction mixture contained 100 µl of acetate buffer (50 mM, pH 5.5) with 100 µl substrate (5 mM). After incubation at 37 ˚C for 15 min, the absorbance at 410 nm (ε410= 18.3 $mM^{-1}cm^{-1}$) was measured (microplate reader, Bio-RAD, USA). One unit (U) of acid phosphatase activity was defined as the amount of enzyme releasing 1µmol of pNP min-1. The assays for leucine aminopeptidase and cellobiohydrolase activity followed the same protocol with adjustments in incubation time, temperature, buffer pH and the substrate concentration (Table S2). For laccase activity, the protocol was as follows: the mycelial samples were placed in wells of a 96-well microplate with 100 µl of acetate buffer (pH 5) and 100 µl of 2,2'- azinobis-3-ethylbenzothiazoline-6-sulfonate (ABTS) solution (2 mM). After incubating at 25 ˚C for 25 min, the mycelia were removed from the plate and the oxidization of ABTS was monitored by determining the absorbance at 405 (ε405 = 36.8 $mM^{-1}cm^{-1}$) with a plate reader. One unit of laccase activity was defined as the amount of enzyme required to oxidize 1 µmol of ABTS min-1. The final enzyme activity was standardized by the dry weight of mycelial sample, expressed as U $mg^{-1}_{(dw)}$.

Table S3. Conditions of enzyme activity tests including concentration of substrates, incubation time, incubation temperature, buffer systems and pH value.

Tested enzyme (Enzyme commission number)	Substrate/ concentration (mM)	Time (min)	Tempera-ture (°C)	Buffer	pH
Laccase (1.10.3.2)	ABTS/ 2	15	25	Acetate buffer	5.0
Cellobiohydrolase (3.2.1.91)	pNP-cellobioside/ 2	120-240	37	Acetate buffer	5.5
Leucine aminopeptidase (3.4.11.1)	Leucine-p-Nitroanilide/ 5	30	50	Tris buffer	8.0
Acid phosphatase (3.1.3.2)	pNPP/ 5	15	37	Acetate buffer	5.5

4. Phylogenetic signal in architectural traits

Table S4. Phylogenetic signal estimated by K statistics.

Trait	K	P-value
K_r	0.038	0.895
Lac	0.341	0.157
Cel	0.418	0.022
Leu	0.627	0.006
Pho	0.235	0.235

5. The Comparison of the traits at phylum-level

To compare the difference of traits at phylum-level, the data were tested by analysis of variance and TukeyHSD test. The statistics are summarized in Table S5.

Table S5. Summary of Anova and TukeyHSD.

Trait	Anova		TukeyHSD test's p-value		
	F	p-value	B-A	M-A	M-B
Kr	6.49	0.0048	n.s.	0.0034	n.s.
Lac	13.02	0.0001	0.0004	n.s.	0.0001
Cel	5.79	0.0069	n.s.	0.0049	n.s.
Leu	5.49	0.0097	n.s.	0.0080	n.s.
Pho	1.28	0.2950			

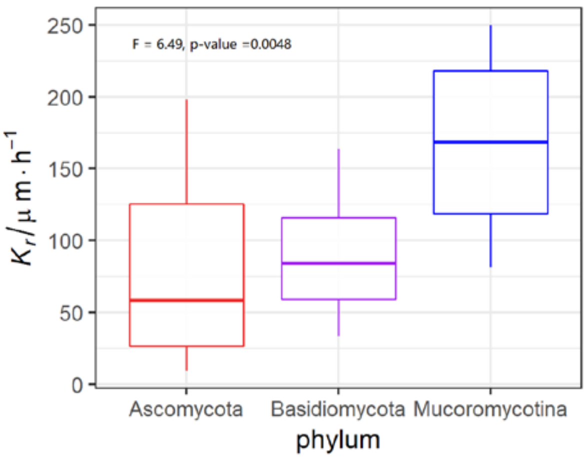

Fig. S2. K_r comparison at phylum-level. Pairwise comparison: B-A: not significant; M-A: p = 0.0034; M-B: not significant.

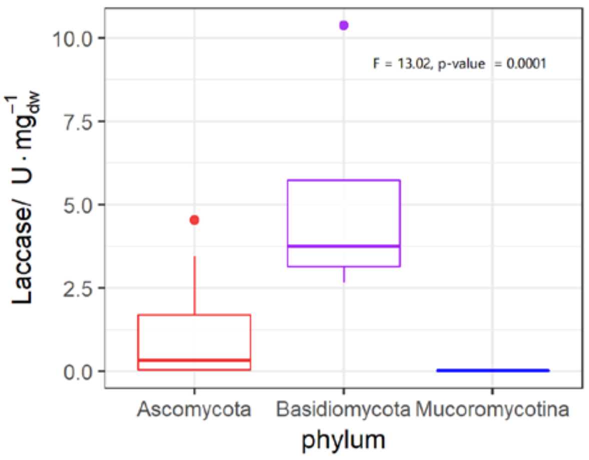

Fig. S3. Laccase activity comparison at phylum-level. Pairwise comparison: B-A: p=0.0004; M-A: not significant; M-B: p < 0.0001.

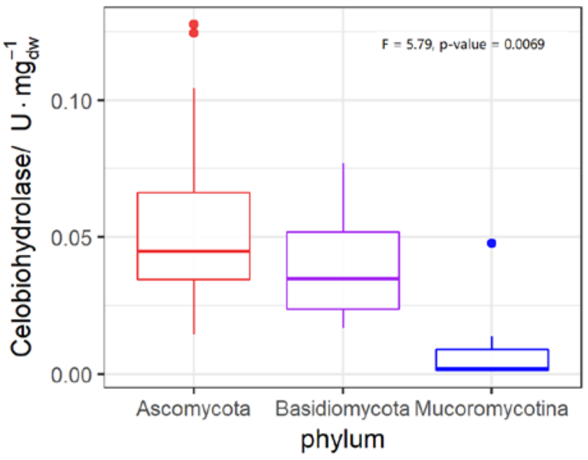

Fig. S4. Cellobiohydrolase activity comparison at phylum-level. Pairwise comparison: B-A: not significant; M-A: p = 0.0049; M-B: not significant.

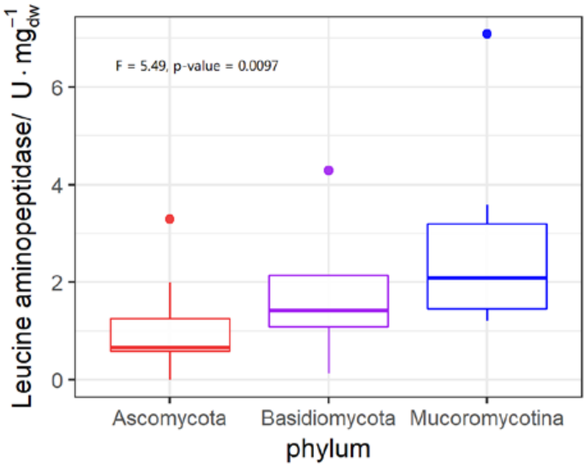

Fig. S5. Leucine aminopeptidase activity comparison at phylum-level. Pairwise comparison: B-A: not significant; M-A: p = 0.0077; M-B: not significant.

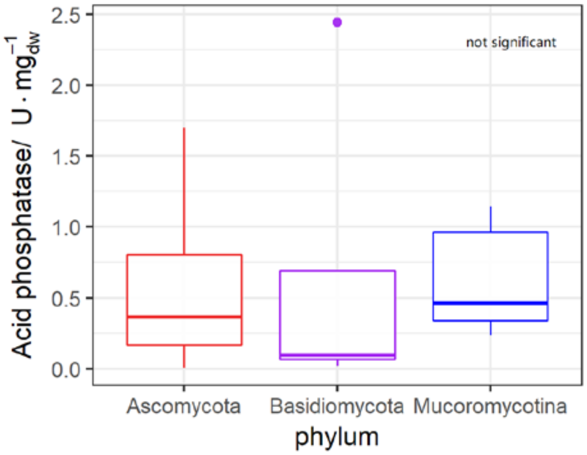

Fig. S6. Acid phosphatase comparison at phylum-level.

6. Regression between K_r and the enzyme activities.

Linear regression and quantile regression are applied between K_r and each of the enzyme activities one by one. Table S6. gives the statistical summary of the tests.

Table S6. Statistics of linear and quantile regression between single enzyme activity and growth rate, n = 31. The quantiles were set as 0.1, 0.25, 0.5, 0.75 and 0.9, and the significant quantiles are listed in the table.

K_r ~ Enzyme	Linear regression model			Quantiles regression model	
	F-statistic	P-value	R_{adj}^2	Significant quantiles	P-value
Lac	4.04	0.054	0.09	0.9	0.008
				0.75	0.036
Cel	4.98	0.033	0.11	0.9	0.007
Leu	3.74	0.063	0.08	0.5	0.030
Pho	1.83	n.s.	0.03	n.s.	

n.s.: not significant

References

Lehmann, A., Zheng, W., Soutschek, K. & Rillig, M. C. How to build a mycelium: tradeoffs in fungal architectural traits. Scientific Reports, doi: 10.1038/s41598-019-50565-7.
